# Antibody-targeted paclitaxel loaded nanoparticles for the treatment of CD20^+^ B-cell lymphoma

**DOI:** 10.1038/srep45682

**Published:** 2017-04-05

**Authors:** Wendy K. Nevala, John T. Butterfield, Shari L. Sutor, Daniel J. Knauer, Svetomir N. Markovic

**Affiliations:** 1 Department of Oncology Research, Mayo Clinic, 200 1st St SW Rochester, MN 55905, USA

## Abstract

We developed a nano-antibody targeted chemotherapy (nATC) delivery strategy in which tumor specific and clinically relevant antibodies (rituximab, anti-CD20) are non-covalently bound to the albumin scaffold of nab-paclitaxel (ABX). We define the nanoparticle formed when the 2 drugs are bound (AR160). The newly created nATC retains the cytotoxicity of ABX and CD20 affinity of rituximab *in vitro*. We describe the binding characteristics of the ABX and rituximab in AR160 using peptide mapping/Biacore approach. Flow-based methods, including ImageStream and nanoparticle tracking, were used to characterize the AR160 particles *in vitro*. A mouse model of human B-cell lymphoma was utilized to test *in vivo* efficacy of AR160 therapy, which suggested improved tumor targeting (biodistribution) as the most likely mechanism of AR160 therapeutic superiority over ABX or rituximab alone. These data suggest a novel platform for nATC delivery using a slight modification of existing cancer drugs with significantly improved treatment efficacy.

Non-Hodgkin’s lymphoma (NHL) includes more than 60 distinct types of malignancies with nearly 85% being B-cell lymphomas, most of which are CD20^+^ 1,2,3,4. In 2016 approximately 73,000 new cases of NHL are expected in the US with nearly 21,000 deaths^5^. There are two general types of NHL, indolent and aggressive^6^, which both respond well to first line therapy with an objective response rate of about 50 to 70% with chemotherapy or rituximab alone^7^,^8^,^9^ and 75 to 90% when chemotherapy is combined with rituximab^10^,^11^. Primary refractory and relapsed lymphomas remain difficult to treat and most of these patients succumb to their disease. Thus, salvage therapy for refractory/relapsed B cell NHL remains an unmet need in cancer therapy, especially after stem cell transplant^12^.

Balancing tumor efficacy with drug toxicity continues to be a major challenge of cancer therapy. Antibody drug conjugates (ADC) have been an attractive and sometimes effective option to maximize therapeutic index. Gemtuzumab ozogamicin, a CD33 specific ADC, for relapsed acute myeloid leukemia^13^,^14^ and brentuximab vedotin, a CD30 specific ADC, have been commonly used in clinical practice and achieved an 86% overall response rate in patients with relapsed anaplastic large cell lymphoma (ALCL)^15^. Both strategies target a cell bound surface molecule expressed on cancer cells for the given hematological malignancies. In a similar vein, a rituximab/CD20 directed chemotherapy seems a viable therapeutic option for B-cell NHL as nearly 95% of all B-cell lymphomas are CD20^+^ 16,17, and rituximab given in addition to chemotherapy provides improved clinical outcomes over chemotherapy alone^10^,^18^,^19^,^20^,^21^.

Previously we have shown that, due to the unique aspects of the manufacturing of therapeutic monoclonal antibodies and nab-paclitaxel (ABX), humanized therapeutic monoclonal antibodies, including bevacizumab, trastuzumab and rituximab, bind with a high affinity to ABX offering the ability to specifically target the chemotherapeutic agent within ABX, paclitaxel, to the tumor. Furthermore, we have demonstrated that bevacizumab coated ABX (AB160) was more efficacious than ABX alone in a mouse model of human melanoma^22^. Herein, we present our results testing rituximab coated ABX (AR160) for the treatment of B-cell NHL in a preclinical model of lymphoma. Our data suggests that the therapeutic superiority of AR160 is primarily driven by favorable bio-distribution of AR160 into the tumor relative to ABX or rituximab alone. These data were the foundation for the clinical development of AR160, currently in progress.

## Results

### AR160 particle content and binding

**Figure 1:**
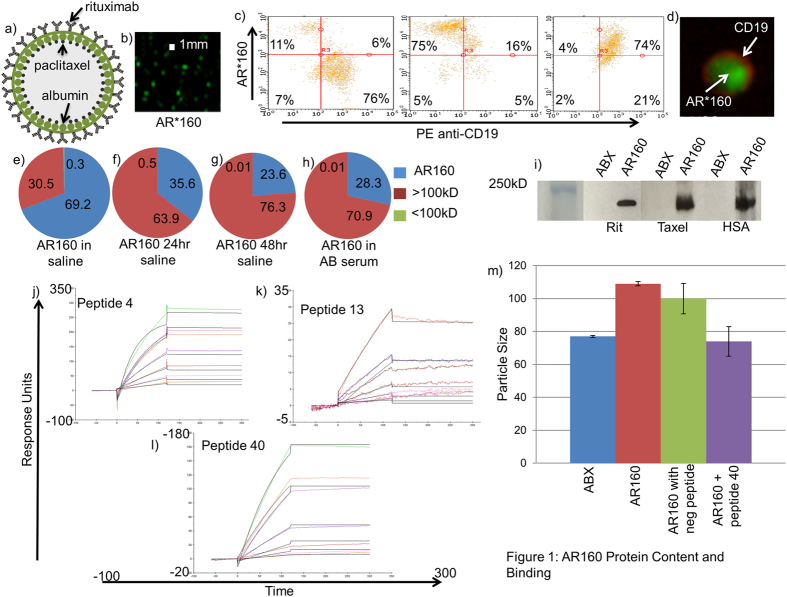
ImageStream and Western blot characterization of AR160. (**a**) A schematic representation of AR160. (**b**) Rituximab was tagged with AlexaFluor 488 and co-incubated with ABX for visualization. ImageStream reveals fluorescently labeled nanoparticles of approximately 0.1 uM. (**c**) ABX was tagged with AlexaFluor 488 and co-incubated with rituximab and Daudi cells were stained with either PE anti-human CD19, fluorescent AR160, or both. Cells were analyzed by Guava flow cytometery. Daudi cells were about 75% positive for CD19, AR160 or both. (**d**) The labeled Daudi cells were also run by ImageStream and an image of a doubly stained Daudi cell is shown. (**e**) AR160 was separated into 3 fractions: the particulate, and proteins greater than and less than 100 kD. Paclitaxel concentration in each fraction was determined by HPLC and showed about 69.2% of paclitaxel is in the particulate and the remaining paclitaxel is among proteins greater the 100 kD. Paclitaxel was measured in AR160 fractions after 24 (**f**), 48 hours (**g**), and 60 minutes in AB serum (**h**). Data shows a shift of the majority of paclitaxel from the particulate to the proteins >100 kD. (**i**) Western blot was performed on the greater than 100 kD fraction and rituximab, paclitaxel and albumin co-localized in a band of approximately 200 kD. Biacore screening of an albumin peptide library for binding to riruximab reveals 3 binding peptides (**j**) HSA peptide 4 binds to rituximab with a Kd of 5.7 × 10^−8^. (**k**) HSA peptide 13 binds to rituximab with a Kd of 4.0 × 10^−7^. (**l**) HSA peptide 40 binds to rituximab with a Kd of 5.2 × 10^−10^. (**m**) ABX was incubated for 30 minutes with 4 mg/ml rituximab and either no peptide (AR160), 10x molar excess, relative to antibody, of control peptide (ABX+Rit+control) or HSA peptide 40 (ABX+Rit+HSA peptide 40). Particle sizes were determined by nanoparticle tracking analysis utilizing the NS300. HSA peptide 40 was shown to block the formation of AR160 suggesting the peptide blocks rituximab from binding ABX. Results are representative of 3 experiments.

Previously we have shown that rituximab binds ABX at high affinity with a dissociation constant in the picomolar range, and when 4 mg/ml of rituximab is mixed with 10 mg/ml ABX a 160 nm nanoparticle is formed, AR160 (Fig. 1a)^22^. In order to visualize the AR160 nanoparticles we labeled rituximab with AlexaFluor 488 and incubated the labeled rituximab with 10 mg/ml ABX. AR160 nanoparticles containing labeled rituximab were visualized using Amnis ImageStream flow cytometry (Fig. 1b).

To determine AR160 binding to membrane bound CD20, Daudi cells were stained with PE-anti-human CD19 and AR160, which contained Alexa fluor labeled ABX coated with rituximab. Scatterplots show a population of Daudi cells that are 75% positive when stained with PE anti-human CD19, 75% positive when stained with fluorescently tagged AR160, and 74% double positive when stained with both PE anti-human CD19 and labeled AR160 suggesting that AR160 binds Daudi cells with protein specificity (Fig. 1c). Stained Daudi cells were also visualized by imaging flow cytometry with the Amnis ImageStream (Fig. 1d).

We quantitated paclitaxel in AR160 fractions that included the spun particulate, and proteins greater and less than 100 kD. We measured paclitaxel in these fractions for freshly prepared AR160 (Fig. 1e), 24-hour AR160 in saline (Fig. 1f), 48-hour AR160 in saline (Fig. 1g) and 24-hour AR160 added to AB serum for 1 hour (Fig. 1h). We found that about 70% of the paclitaxel remained with the AR160 particulate and most of the remaining 30% was with proteins greater than 100 kD with a very small percentage (0.3%) being in the less than 100 kD protein fraction (Fig. 1e). After 24 and 48 hours in saline there was a shift of paclitaxel from the particulate into the fraction with proteins greater than 100 kD with 2/3 being in the greater than 100 kD fraction and 1/3 in the particulate fraction with no increase of paclitaxel into the less than 100 kD fraction. This data suggests that as the large particle dissociates the paclitaxel is bound to proteins, which could be rituximab and albumin. The paclitaxel content after a 1 hour incubation in AB serum stayed about the same as the 24 hours in saline (63.9 vs 70.9%) suggesting that the greater than 100 kD fraction is stable in serum and the paclitaxel is not exchanged from the ABX albumin to endogenous albumin, which is crucial to maintain the antibody-ligand mediated targeting of tumor cells. To confirm the contents of the fractions we did Western blot analysis by staining for paclitaxel, rituximab and human albumin. We found that the two proteins and paclitaxel co-localize in a band that is approximately 200 kD (Fig. 1i) in the AR160 particulate and >100 kD fractions with minimal protein in the <100 kD fraction, further suggesting that the 160 nm particle dissociates into functional units that retain tumor targeting ability and contains paclitaxel within the 200 kD macromolecular species.

We utilized Biacore surface plasmon resonance technology to determine where the binding sites between rituximab and ABX were located. An albumin peptide library of 18 amino acid peptides was constructed, and each albumin peptide was run against rituximab fixed on a CM5 chip. We found 3 albumin peptides that bound rituximab: peptide 4, peptide 13, and peptide 40 with Kd of 5.7 × 10^−8^ (Fig. 1j), 4.0 × 10^−7^ (Fig. 1k), and 5.2 × 10^−10^ (Fig. 1l), respectively. Interestingly, peptide 40 maps to the well-characterized Sudlow II site of albumin, a known hydrophobic binding site that binds many drugs^23^,^24^.

Previously we have shown that co-incubation of 4 mg/ml rituximab with 10 mg/ml of ABX results in a shift of size of the 130 nm ABX alone to approximately 160 nM when rituximab is bound to ABX as determined by Malvern Mastersizer^22^. By Nanosight, we observed the same 30 nm shift in size, with the size of ABX alone being about 80 nM and AR160 size of about 110 nM (Fig. 1m). We used the albumin binding peptides in a 10X molar excess to see if they would interfere with the formation of AR160. We found the size of ABX alone to be 77 nm, while rituximab binding to ABX yielded 100 nm particles. When the albumin binding peptide 40 was added to an incubation of 10 mg/ml ABX and 4 mg/ml rituximab, the resultant nanoparticle was 70 nm, the size of ABX alone, suggesting that peptide 40 effectively inhibited the formation of AR160 while a non-binding control peptide resulted in 100 nm particles, the size of AR160 (109 nm) in this experiment (Fig. 1m). The results with the binding peptides 4 and 13 demonstrated a more heterogenous nanoparticle population suggesting that these 2 albumin peptides, which have less affinity for rituximab, incompletely blocked the formation of AR160 (Supplementary Fig. 2).

### *In vitro* testing of AR160

**Figure 2:**
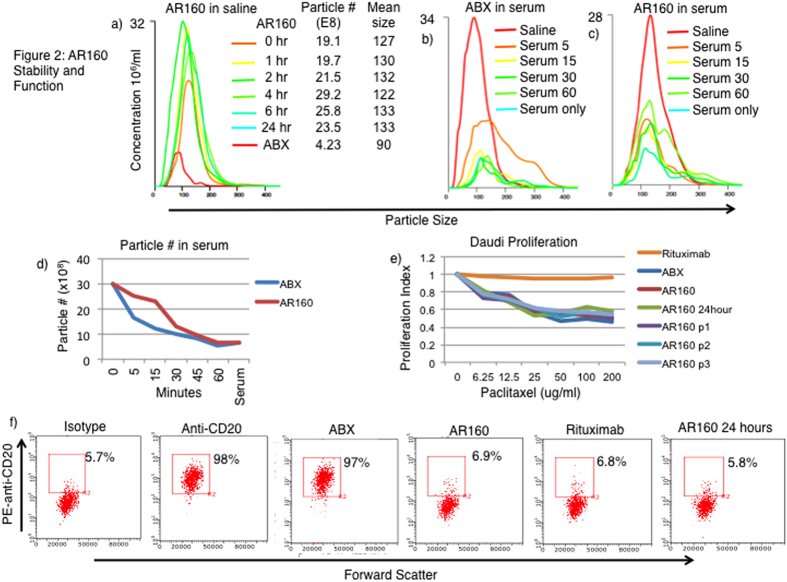
AR160 maintains paclitaxel’s toxicity and rituximab’s biding selectivity while increasing particle stability. (**a**) ABX and AR160 was prepared and incubated for 24 hours in saline at room temperature and size and particle number was measured at 0, 1, 2, 4, 6, and 24 hours by NS300 nanoparticle tracking system. AR160 remained stable over 24 hours as particle size and number remained unchanged during that time. The particle size distributions and particle size and numbers are shown. (**b**,**c**) ABX and AR160 were prepared, and 30 × 108 particles were added to human AB serum and incubated for 60 minutes in human AB serum. Particle size and numbers were determined at 5, 15, 30, and 60 minutes after being added to the AB serum. Particle size distributions for ABX (**b**) and AR160 (**c**) are shown. (**d**) Graphical representation of the particle number of ABX relative to AR160 after incubation in AB serum. (**e**) To verify toxicity of AR160, we treated CD20+ Daudi cells with AR160, ABX and rituximab. Cells were treated overnight with the drugs at concentrations from 0–200 ug/ml with the addition of EdU, a thymidine analog. The level of proliferation was determined by staining cells with FITC labeled anti-EdU. The proliferation index was calculated by normalization to the untreated positive control. (**f**) Rituximab ligand binding was confirmed by flow cytometry in which the Daudi cells were pretreated with rituximab, ABX, and AR160 and then stained with PE anti-human CD20. The drug-pretreated samples were compared to isotype control and PE anti-human CD20 alone serving as negative and positive controls, respectively. Rituximab and AR160, but not ABX alone blocked subsequent PE anti-human CD20 binding. Data is representative of 3 experiments.

We tested the stability of AR160 relative to ABX alone using Nanosight technology. ABX and AR160 were made as described and allowed to sit at room temperature for 0 to 6 and 24 hours and the particle quantity and size were measured at each time point (Fig. 2a). The number of particles of ABX alone was 4.23 × 10^8^ compared to AR160, which had a range of 19.1 to 23.5 × 10^8^ particles at 0–24 hours. The mean size of ABX was 90 nm and much smaller than AR160 that had mean sizes of 127–133 nm at 0–24 hours. Additionally the size of AR160 was stable through 24 hours as suggested by the number and size of the particles remaining the same throughout the incubation time. ABX became unstable during the incubation time and difficult to measure particle size and number so only time 0 is shown (Fig. 2a).

To access the stability of AR160 relative to ABX in serum, we added the equivalent number (30 × 10^8^) of particles of ABX and AR160 to human AB serum and quantitated the number of particles remaining at 5, 15, 30 and 60 minutes of incubation at room temperature (Fig. 2b and c). At each time point more AR160 particles were measured (19, 16, 11 and 10 × 10^8^) relative to ABX alone (11, 6.6, 4.2, and 5.2 × 10^8^) at 5, 15, 30, and 60 minutes, respectively (Fig. 2d). Furthermore, ABX particle number returned to baseline relative to serum only after 15 minutes of incubation while AR160 particle numbers remained higher throughout the 60-minute incubation than the 6.3 × 10^8^ particles measured in serum only (Fig. 2d).

To ensure that the paclitaxel in AR160 maintained its anti-proliferative capacity, we tested AR160 and 24-hour old AR160, relative to ABX and rituximab alone, in an *in vitro* toxicity assay using Daudi cells. The cell proliferation was measured with EdU, a thymidine analog, that was detected with a FITC conjugated anti-EdU antibody and enumerated by flow cytometry. The IC50 of all the paclitaxel containing drugs was about 25 ug/ml while rituximab alone was not toxic (Fig. 2e); therefore, paclitaxel toxicity is not compromised in the context of AR160.

We tested the ligand binding capability of the rituximab in the context of the AR160 complex. To do this, we incubated CD20^+^ Daudi cells with rituximab, ABX, AR160 and 24-hour old AR160. After incubation the cells were washed and stained with PE-mouse anti-human CD20 and enumerated by flow cytometry. An isotype control (5.7% positive) and PE-mouse anti-human CD20 (96% positive) were used as negative and positive controls, respectively. The results show that rituximab and AR160 prohibit subsequent binding of the anti-human CD20 antibody suggesting that rituximab alone and in the context of the AR160 complex bind CD20, thereby, inhibiting the binding of the anti-CD20 antibody. ABX alone did not inhibit binding of the antibody indicating that the rituximab in the AR160 particle retains its ligand binding properties in a specific manner (Fig. 2f).

### *In Vivo* Testing of AR160

**Figure 3:**
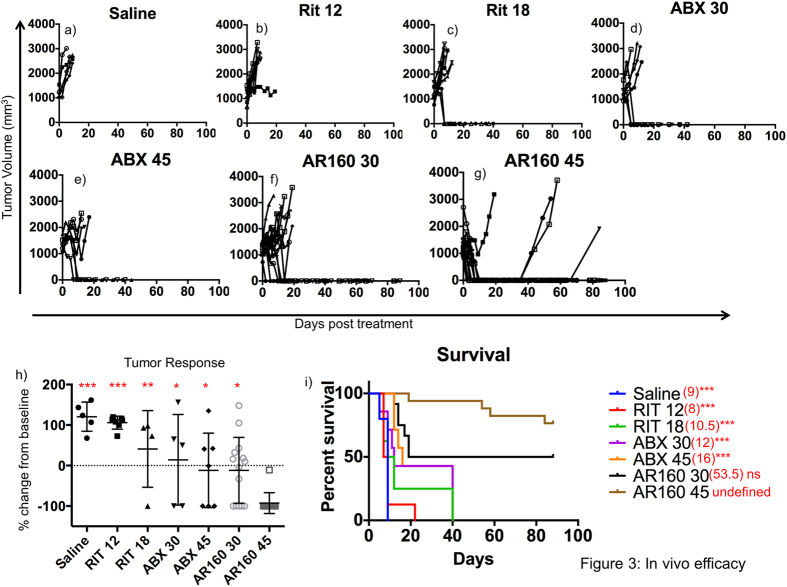
AR160 has improved tumor efficacy relative to ABX and rituximab alone. Median survival is extended in mice treated with AR160 with 13/17 mice having a durable, complete response. (**a**–**f**) After treatment with saline, 12 mg/kg rituximab (Rit 12), 18 mg/kg rituximab (Rit 18), 30 mg/kg ABX (ABX 30), 45 mg/kg ABX (ABX 45), AR160 at the lower doses (AR160 30) and AR160 at the higher doses (AR160 45), tumor growth was monitored 2–3 times/week and growth kinetics are shown. (**h**) Tumor response was calculated at day 10 by {(tumor size on day of treatment) − tumor size on day 10 (or day of death)/tumor size on day of treatment} * 100. P-values were determined by student’s t-test: p = <0.0001(***), p = <0.001 (**), and p = <0.01 (*). (**i**) Kaplan-Meier curves were generated and median survival was determined. The difference in median survival between AR160 45 and saline, Rit 12, Rit 18, ABX 30, ABX 45 were all significant with p values = <0.0001. The difference in survival between AR160 45 and AR160 30 was not significant with a p value = 0.0715. Similar results were seen in 3 separate mouse experiments.

A xenotransplant model of B-cell lymphoma, Daudi cells, was established in athymic nude mice. Once tumors were established, mice were treated with saline, 12 mg/kg rituximab (RIT12), 18 mg/kg rituximab (RIT18), 30 mg/kg ABX (ABX 30), 45 mg/kg ABX (ABX 45), or AR160 containing either 12 mg/kg rituximab and 30 mg/kg ABX (AR160 30) or 18 mg/kg rituximab and 45 mg/kg ABX (AR160 45). Mouse tumors were measured 2–3 times/week and tumor growth kinetics were determined (Fig. 3a–g). On day 10 post treatment tumors were measured and the percent change in tumor size from baseline was calculated. By day 10, all the mice in the AR160 45 treated group (17/17) had a tumor response while 94.1% (16/17) had complete tumor responses compared to 7/14 (50%), 3/7 (42.8%), 1/4 (25%), 0/7 (0%) and 0/5 (0%) of mice having responses in the AR160 30, ABX 45, ABX 30, RIT 18, RIT 12, and saline groups, respectively (Fig. 3h). The percentage of mice alive at day 10 in each group were 0%, 12%, 38%, 43%, 71%, 92% and 100% for saline, RIT 12, RIT 18, ABX 30, ABX 45, AR160 30, and AR160 45 groups, respectively (Fig. 3h). At day 10 post treatment for each mouse the change from baseline was calculated by {tumor size (day 0) − tumor size (day 10)}/tumor size (day 0)} * 100. These changes in baseline were plotted for each mouse (Fig. 3h) and pairwise statistical analysis was performed between the AR160 45 group and each other treatment group individually by unpaired student’s t-test. The difference in change from baseline was statistically significant between the AR160 45 group and all other groups; p = <0.0001 for saline and RIT 12, p = 0.0003 for RIT 18, p = 0.0054 for ABX 30, p = 0.0098 for ABX 45, and p = 0.003 for AB160 30. The median survival of mice treated with AR160 45 remained undefined at 90 days when all mice were sacrificed compared to 9, 8, 10.5, 12, 16 and 53.5 days for mice treated with saline, RIT 12, RIT 18, ABX 30, ABX 45 and AB160 30, respectively (Fig. 3i). The median survival for the AR160 45 group was significantly higher than mice in the saline, RIT 12, RIT 18, ABX 30, ABX 45 (p = <0.0001) groups while the difference between the 2 AR160 groups was not significant (p = 0.0715).

### *In vivo* imaging

**Figure 4:**
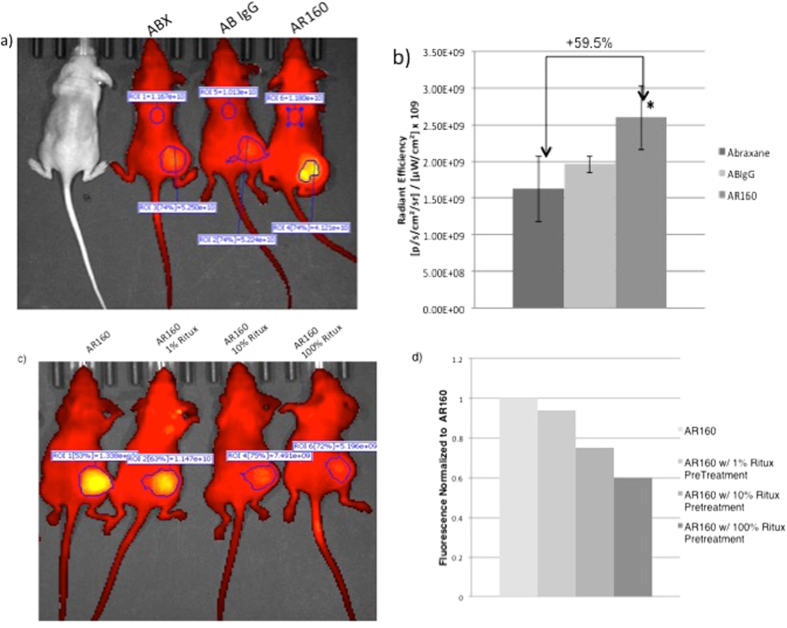
Treating mice with AR160 compared to ABX or ABX with IVIG attached results in increased drug deposition in the tumor. The increased drug deposition in the tumor is antibody-ligand mediated. (**a**) ABX was labeled with AlexaFluor 750, then bound to either to IVIG as a negative control or rituximab (AR160) and Perkin Elmer IVIS Spectrum was employed to fluorescently quantify the concentration of ABX in each tumor. (**b**) Regions of interest (ROI) were made for the tumor and a distal area on the back as background. The background ROI for each mouse was subtracted from the tumor ROI and the resultant radiant efficiency were graphed and about 60% more drug was seen in tumors treated with AR160 relative to ABX alone and IVIG bound ABX. (**c**) Mice were treated with fluorescently tagged AR160 after either no pretreatment or pretreatment with 1%, 10% or 100% dose of rituximab 24 hours prior to AR160 injection. Pretreatment with rituximab blocks AR160 deposition in a dose-dependent manner. (**d**) Similar ROI were generated and background was subtracted. The fluorescence was normalized to the AR160 treatment group. The images are representative of 3 experiments with 3 to 5 mice/treatment group.

We hypothesized that the reason for the improved tumor efficacy of AR160 relative to ABX was increased deposition of chemotherapy drug in the tumor of mice treated with AR160 as suggested in our earlier paper testing bevacizumab bound ABX for melanoma^22^. In order to test our hypothesis, we employed *in vivo* fluorescence imaging with the Perkin Elmer IVIS Spectrum. ABX was labeled with Alexa fluor 750 and bound to either rituximab or IVIgG as a negative control, and images were taken at 24 hours (Fig. 4a). Background fluorescence was measured using a region of interest (ROI) on the mouse back and subtracted from the fluorescence measurement in the tumor ROI. After the background was subtracted, a 59.5% increase was detected in mice given AR160 relative to ABX alone and ABX bound IVIgG (Fig. 4b) suggesting that addition of the lymphoma-targeting antibody, rituximab, increased deposition of the chemotherapy at the tumor site. To show that the increased tumor deposition of ABX in the AR160 treated mice was antibody-ligand mediated, we pretreated mice with 1% (0.12 mg/kg), 10% (1.2 mg/kg) and 100% (12 mg/kg) of the rituximab dosage in AR160 24 hours prior to injecting the fluorescently label AR160. We imaged the mice at 24 hours post AR160 injection (Fig. 4c). The mice injected with AR160 alone had a high level of fluorescently labeled AR160 in the tumor while the pretreatment with increasing amounts of rituximab showed diminished quantities of labeled AR160 in the tumors (Fig. 4d). Taken together this data suggests that there are increased levels of labeled AR160 at the tumor site relative to ABX alone, and this increase of drug deposition in the tumor is mediated by the CD20 ligand specificity of the rituximab.

## Discussion

First line chemotherapy is clinically effective for the treatment of NHL^7^,^8^,^9^ with additional benefit if rituximab is added to chemotherapeutic regimens^10^,^25^. However, relapsed and refractory lymphomas remain more difficult to treat and many of these patients succumb to their disease^12^. In an effort to provide more treatment options for these patients, we developed a nano-antibody targeted chemotherapy (nATC) platform by non-covalently binding rituximab to the albumin scaffold of ABX. We have characterized the binding of the two drugs and determined the sites where the albumin protein binds to rituximab. We have shown the individual drugs maintain their function in the context of the nanoparticle, AR160. We tested AR160 in a xenotransplant model for human B-cell lymphoma and showed improved tumor efficacy relative the drugs individually. Finally, we demonstrated by *in vivo* imaging that the improved drug efficacy was due to higher drug deposition in the tumor, which was mediated by the antibody interaction with the tumor-expressed ligand.

Historically, the standard first line therapy for NHL has been various combinatorial chemotherapy regimens such as: cyclophosphamide, doxorubicin, and prednisone (CHOP), cyclophosphamide, vincristine, and prednisone (CVP), or mitoxantrone, chlorambucil, and prednisone (MCP)^2^. More recently, the monoclonal anti-CD20 antibody, rituximab, has been added to these chemotherapy regimens and provided additional clinical benefit relative to chemotherapy alone^10^,^26^,^27^,^28^. In elderly patients with diffuse large B-cell lymphoma (DLBCL), rituximab in addition to CHOP therapy improved overall response rates from 63% to 73%^21^ with similar results in young patients with DLBCL^29^. In aggressive follicular lymphoma (FL) the addition of rituximab to chemotherapy showed improved response rate, progression free and overall survival^10^,^26^,^27^. A significant survival benefit was also seen in patients with CLL^30^,^31^ and MCL^20^,^32^, when rituximab was added to chemotherapy in both initial treatment and in pretreated patients. Furthermore, there is evidence that rituximab monotherapy may benefit patients with relapsing or refractory lymphoma^33^ with additional benefit when rituximab is given with chemotherapy^19^; therefore, rituximab tumor targeted chemotherapy seems a viable treatment option. Here we show in a human lymphoma mouse model that AR160 had a significantly increased tumor response rate (p = 0.0003 and 0.0098) (Fig. 3h), and median survival (p = <0.0001) (Fig. 3i) relative to rituximab or chemotherapy alone. We believe that AR160 should be tested in the clinical setting for refractory and relapsed NHL and have received IND approval from the FDA (IND#131847).

Human serum albumin, an abundant serum protein, has been extensively studied as a drug carrier due to its ability to bind many different ligands^34^. Several albumin drug carriers are used in the clinical setting to treat diabetes^35^,^36^, rheumatoid arthritis^37^, infectious disease^38^ and cancer^39^. Specifically an albumin bound paclitaxel, ABX, has been used for the treatment of breast^40^ and pancreatic cancer^41^ as well as melanoma^42^. Because of unique aspects of the manufacturing of ABX^43^, in which hydrophobic paclitaxel is encapsulated in albumin making it water soluble, albumin-binding sites are made available to allow non-covalent binding of the therapeutic monoclonal antibody, rituximab. This feature has allowed us to exploit the ABX albumin scaffold to bind tumor-targeted antibodies, thereby increasing tumor drug deposition (Fig. 4) and tumor efficacy (Fig. 3). Using surface plasmon resonance, we identified 3 albumin-binding sites that bind to rituximab. Albumin peptide 40 is contained in the well-characterized Sudlow II site (Fig. 1)^24^. Further understanding of the protein interactions of rituximab and ABX is the subject of intensive investigation in our laboratory.

Nano-antibody targeted chemotherapy, AR160, may be more advantageous than chemotherapy alone or rituximab given with, but sequential to chemotherapy because higher concentrations of the chemotherapy can be targeted specifically to the tumor increasing efficacy while limiting unwanted toxicity, the main goal of antibody targeted cancer therapy^39^,^44^,^45^. Effective ADC has been a long sought after alternative to chemotherapy alone^46^. Although one ADC, brentuximab vedotin, is in clinical use for relapsed and refractory ALCL^47^, ADCs have not had broad clinical application due to limited efficacy and high toxicity^46^,^48^,^49^. One explanation for limited clinical efficacy of ADC to date is that increased drug deposition in the tumor has yet to be proven^50^. We have shown in our mouse model by *in vivo* imaging that AR160 does successfully target the tumor in an antigen specific manner that results in increased levels of ABX in CD20^+^ Daudi tumors (Fig. 4a–d). Furthermore our previously developed VEGF targeted chemotherapy, AB160, has had promising clinical results in phase I clinical (NCI-2013-01782) testing in metastatic ovarian cancer and melanoma.

In conclusion, we have developed a simple way to generate nATC for NHL by binding rituximab to the albumin scaffold of ABX. We have identified the albumin sites to which rituximab binding occurs. In a mouse model of CD20^+^ lymphoma we show a profound anti-tumor effect that is curative in a majority of mice treated with AR160. Furthermore, we have shown that improved tumor efficacy is due to increased antibody-mediated drug deposition in the tumor. Clinical testing of AR160 is warranted to determine the full clinical relevance of this nano-antibody targeted chemotherapy.

## Materials and Methods

### AR160 formation and Nanosight

ABX (Celgene, Summit, NJ) and rituximab (Genentech, San Francisco, CA) were mixed at 10 mg/ml and 4 mg/ml, respectively in 0.9% saline and incubated for 30 minutes at room temperature. For the peptide competition assay, 10 mg/ml of ABX was incubated for 30 minutes with 4 mg/ml of rituximab and a 10 molar excess of a control peptide, HSA Peptide 40, or no peptide. We determined particle size and number by Malvern Nanosight (Malvern, Worcestshire, UK), which uses light scattering and Brownian motion to obtain particle size and concentration.

### Surface Plasmon Resonance

A peptide library of human serum albumin (New England Peptide, Inc Gardner, MA) consisting of 18 amino acid peptides with 6 amino acid overlap was constructed. Peptides were suspended to 5–10 mg/ml in HBS-EP plus running buffer. Water insoluble peptides were dissolved in 10% DMSO (Sigma-Aldrich, St. Louis, MO). Rituximab was immobilized onto Biacore CM5 (GE Healthcare, Chicago, IL) chips via amine coupling. Biacore X-100 (GE Healthcare, Chicago, IL) was used to screen the albumin peptide libraries over immobilized rituximab. Peptides were screened from 1–50 ug/ml with an exposure time of 120 seconds. Biacore X100 software was used to determine binding kinetics.

### Toxicity Assay

Daudi cells (ATCC, Manassa, VA) were cultured in RPMI-1640 with 1% PSG and 10% FBS. Harvested cells were plated at 0.3 × 10^6^ cells/well in 24 well plates. Cells were incubated with ABX and rituximab alone, or AR160. Paclitaxel concentrations from 0 to 200 ug/ml were used and incubated overnight at 37 °C and 5% CO_2_. Proliferation was determined using the Click-iT EdU (Molecular Probes, Eugene, OR) kit. Briefly, 10 mM EdU was added to the wells and incubated overnight with the paclitaxel exposed Daudi cells. The cells were permeabilized with 1% saponin and intercalated EdU was labeled with a FITC-conjugated antibody. The proliferation index was determined by dividing the FITC positive cells from each treatment by the maximum proliferation of untreated EdU labeled cells.

### Flow Cytometry

ABX and rituximab were labeled with Alexa-fluor 488 (Thermo Scientific, Rockford, IL) by incubation with 1 mg of protein for 60 minutes at room temperature and unbound label was removed by size exclusion column chromatography. Daudi cells were incubated for 30 minutes with labeled AR160, PE anti-human CD19 (Clone HIB19), and PE anti-human CD20 (Clone 2H7) (BD Pharmingen, Franklin Lakes, NJ) at 4 °C. The cells were washed 2x in FACS buffer (1x PBS and 0.5% BSA with 0.1% NaAzide). The cells were run and data was collected on a Guava flow cytometer (Millipore, Billerica, MA). Five thousand events were collected and flow cytometery data was analyzed using GuavaSoft software (Millipore, Billerica, MA) and percentages of AR160^+^, CD19^+^ and CD20^+^ cells were determined. Labeled ABX and PE anti-human CD19 (Clone HIB19)/Alexa-fluor 488 AR160 labeled Daudi cells were collected by Amnis ImageStream (Millipore, Billerica, MA). Inspire software (Millipore, Billerica, MA) was used to analyze data and collect pictures.

### Paclitaxel HPLC

Liquid chromatographic separation of paclitaxel was accomplished using an Agilent Eclipse Plus C18 analytical column (4.6 × 250 mm, 5 um-BC), under a mobile phase of 11:9 ratio of water to acetonitrile, with a flow rate of 1.5 ml/min. A standard curve was generated from 0.5–500 ug/ml. Sample injection volume was 10 ul and detection was accomplished at 228 nm with ~16 minute retention time.

### Western Blot

AR160 was prepared and particulate was spun down by centrifugation at 10,000 rpm for 10 minutes. Supernatant protein complexes were denatured and separated by SDS-PAGE gel chromatography. The proteins were transferred to PVDF membrane and blotted for albumin with rabbit anti-human albumin (1:10,000) (Cell Signaling, Danvers, MA), paclitaxel with rabbit anti-taxol (1:10.000) (AbCam, Cambridge, MA), and rituximab with rat anti-rituximab-HRP (1:500) (Bio-rad, Hercules, CA). Goat anti-rabbit IgG HRP (1:2000) (Cell Signaling, Danvers, MA) was used as a secondary antibody for albumin and taxol. The proteins were visualized by ECL substrate (Thermo Scientific, Rockford, IL).

### Tumor efficacy

Mouse experiments were approved by Mayo Clinic IACUC and were performed in accordance to relevant guidelines and regulations under protocol number A5515. An *in vivo* model of B-cell lymphoma was utilized to test tumor efficacy. Five million Daudi cells were implanted into the right flank of athymic nude mice (Harlan Sprague Dawley, Indianapolis, IN). Tumors were allowed to grow to about 800 mm^3^ and then treated with saline, RIT (12 mg/kg), RIT (18 mg/kg) ABX (30 mg/kg), ABX (45 mg/kg), or AR160 (12 mg/kg RIT and 30 mg/kg ABX) or (18 mg/kg RIT and 45 mg/kg ABX) by 100 ul intravenous injection in the tail vein. Tumor size was monitored and tumor volume was calculated by (length * width^2^)/2. Mice were sacrificed when the tumor size was about 2500 mm^3^. The day 10 percent change from baseline was calculated as follows: [(tumor size on treatment day − tumor size on day 10)/tumor size on treatment day]*100. The change from baseline in the AR160 45 group was compared to each other group individually by unpaired student’s t-test using GraphPad Prism software (GraphPad Software, Inc, La Jolla, CA). Kaplan Meier curves were generated and median survival was calculated using GraphPad Prism software (GraphPad Software, Inc, La Jolla, CA).

### Tumor imaging

ABX was labeled with AlexaFluor 750 dye with SAIVI Antibody Labeling kit (Thermo Scientific, Rockford, IL). ABX and dye were incubated for 60 minutes and unbound label was removed by column chromatography. Labeled ABX was concentrated using Amicon filters (Millipore, Billerica, MA) and incubated with IVIG (CSL Berhing, King of Prussia, PA) or rituximab (Genentech, San Francisco, CA) at 10 mg/ml and 4 mg/ml, respectively, for 30 minutes. Particle size was checked by Nanosight confirm AR160 formation. Mice were injected with 100 ul of 2 mg/ml labeled ABX, IVIgG coated ABX, or AR160. Mice were imaged using a Perkin Elmer IVIS Spectrum (Perkin Elmer, Waltham, MA) at 24-hours post injection. Fluorescent imagery was done at an excitation/emission spectrum of 710/760 and regions of interest (ROI) applied using imaging software (Perkin Elmer, Waltham, MA). Tumor delivery was determined by a measure of average radiant efficiency within the tumor area minus background radiance.

## Additional Information

**How to cite this article:** Nevala, W. K. *et al*. Antibody-targeted paclitaxel loaded nanoparticles for the treatment of CD20^+^ B-cell lymphoma. *Sci. Rep.* **7**, 45682; doi: 10.1038/srep45682 (2017).

**Publisher's note:** Springer Nature remains neutral with regard to jurisdictional claims in published maps and institutional affiliations.

## Supplementary Material

Supplementary Information
