# Supplementary material for: Antibody-targeted paclitaxel loaded nanoparticles for the treatment of CD20+ B-cell lymphoma
Source: Sci Rep. 2017 Apr 5;7:45682. doi: 10.1038/srep45682 (PMC5381215; doi:10.1038/srep45682)
Supplement: Supplementary Information [file srep45682-s1.pdf]

Antibody-targeted paclitaxel loaded nanoparticles for the treatment of CD20$^+$ B-cell lymphoma.

Wendy K. Nevala[1], John T. Butterfield[1], Shari L. Sutor[1] Daniel J. Knauer[1], and Svetomir N. Markovic[1*]

[1]Mayo Clinic, Rochester, MN 55905

Corresponding Author, e-mail: markovic.svetomir@mayo.edu

Supplementary Figure 1: Western Blot

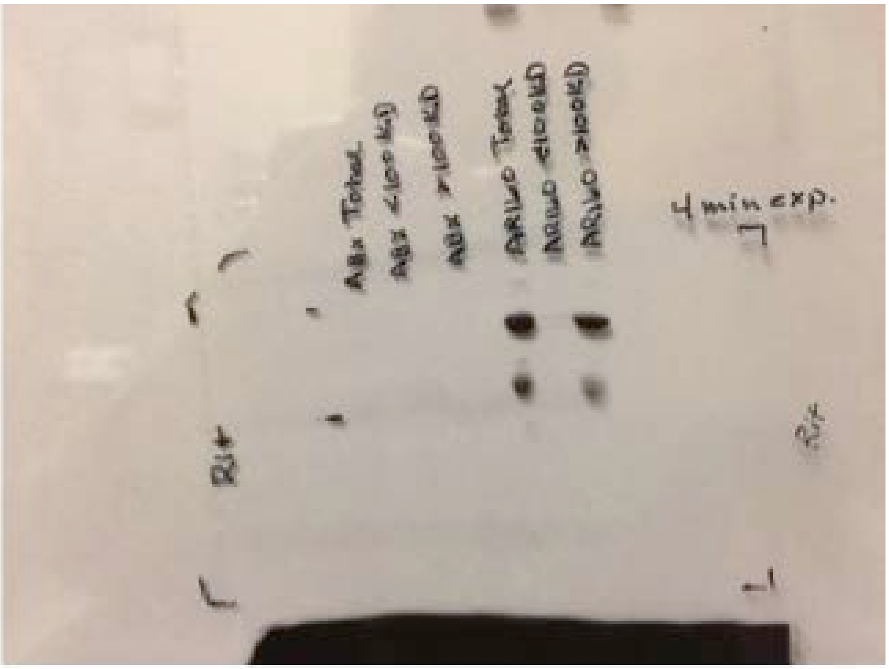

Rituximab Western Blot

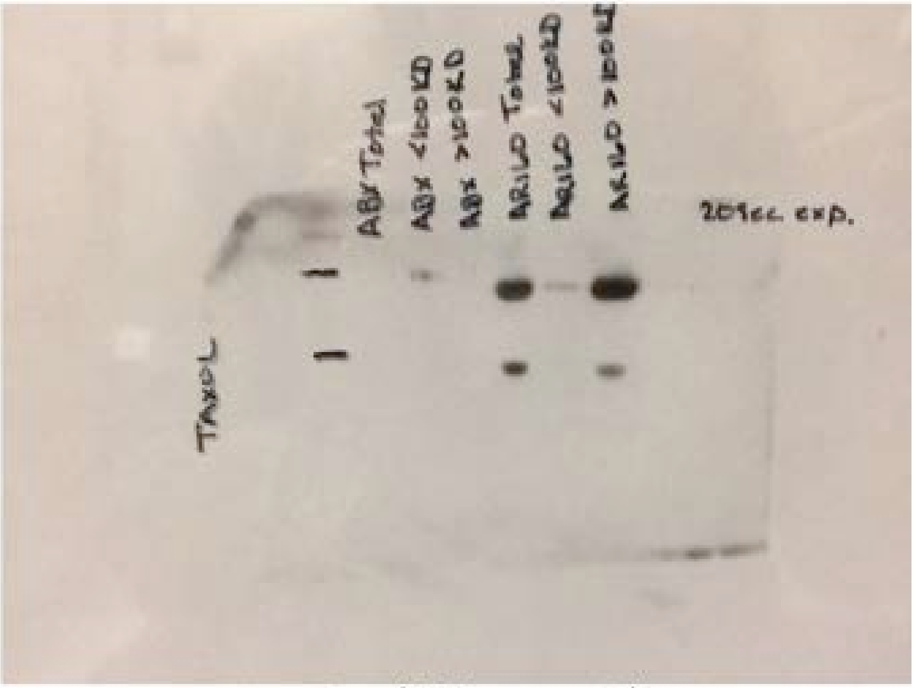

Taxol Western Blot

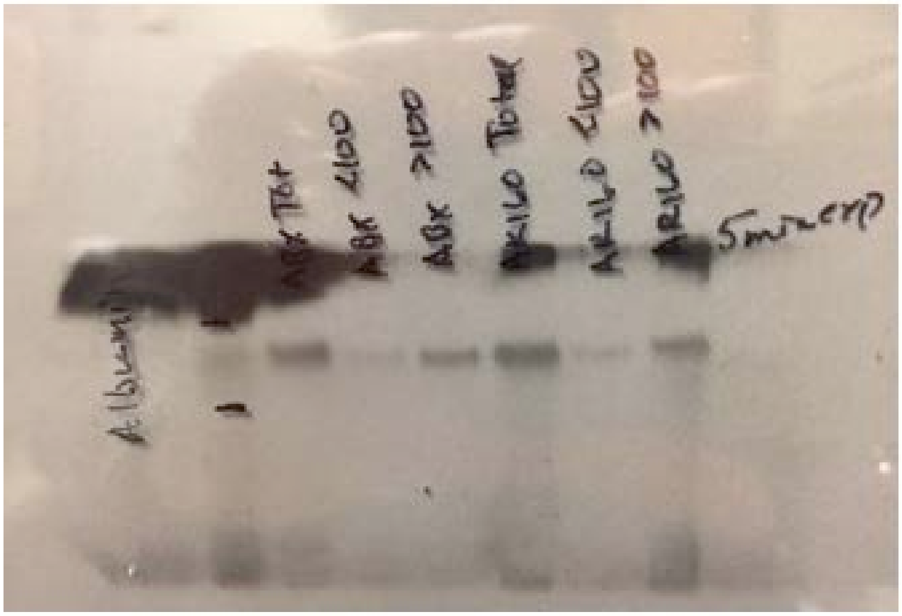

Albumin Western Blot

Supplementary Figure 1: Albumin, rituximab and albumin co-localize in an approximately 200kD band. AR160 was prepared as described and fractionated by filter size exclusion into 3 fractions, the particulate, proteins greater than 100kD and proteins less than 100kD. Western blots were probed for rituximab (rat anti-rituximab HRP 1:500) (a), paclitaxel (rabbit anti-taxol 1:10,000) (b) and albumin (rabbit anti-human albumin 1:10,000) (c). For paclitaxel and albumin goat anti-rabbit IgG HRP was utilized for detection at 1:2000.

Supplemental Figure 2: Peptide inhibition of AR160 formation with HSA peptide 4 and 13.

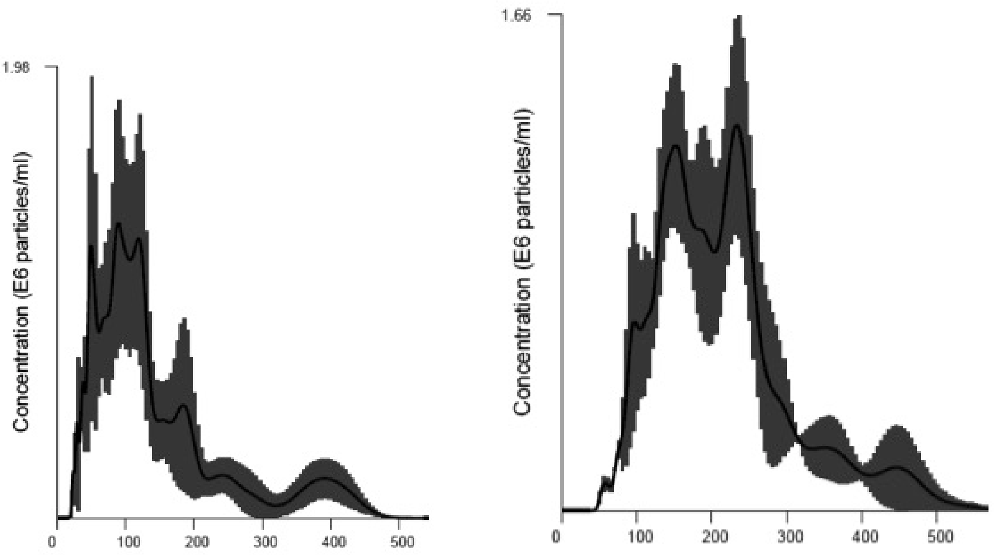

HSA peptide 4
96 +/- 23nm

HSA peptide 13
180 +/- 26nm

Supplemental Figure 2: HSA peptide 4 and 13 incompletely block formation of AR160 particles. A 10M excess of peptide 4 and 13 were added to 4mg/ml rituximab and 10mg/ml ABX and incubated for 30 minutes at room temperature. Nanosight was utilized to determine the size and number of the resultant nanoparticles. Results demonstrate that the resulting particles are heterogenous in size indicating that these peptides only partially inhibited the formation of AR160.
